# 

**Published:** 1893-06

**Authors:** 


### Notice—Postponement

GRAND RAPIDS, April 26, 1893.

At a meeting of the Board of Directors of the Michigan Dental Association held at Detroit in March, owing to the many attractions elsewhere, and the majority of the members of the Association being in favor, it was decided to postpone our regular annual meeting, which was to take place at Ann Arbor in June, for one year.

J. WARD HOUSE,
Sec'y M. D. A.

### NOTICES.

THE Committee of Arrangements for the forty-fourth annual meeting of the American Medical Association, to be held in Milwaukee, June 6, 7, 8, 9, 1893, desire to make the following announcement:

The Western States Passenger Association has made arrangements for the usual reduction of fares to and from the place of meeting—namely, one and one-third rates for the round trip.

By purchasing tickets to Milwaukee, and asking at the time a certificate from the railroad agent, and having that signed by the permanent secretary, Dr. W. B. Atkinson at Milwaukee, return tickets at the reduced fare can be obtained. This arrangement will not, however, admit of any stop-over, and for those who may desire to visit the Columbian Exposition at Chicago, the Committee would recommend that delegates and members purchase their tickets to Chicago and return at Exposition rate, which undoubtedly will be as low, if not lower than the one and one-third rates, and will be good for the season.

Delegates and members should then procure in Chicago round trip tickets, good for the season, between Chicago and Milwaukee, which can be purchased at reduced rates, the tickets now being $4.00 for the round trip, either by rail or steamboat.

The distance is eighty-five miles. Trains run every hour or two, either by the Chicago, Milwaukee & St. Paul or by the Chicago and Northwestern line. Boats leave Chicago at night and in the morning, arriving at Milwaukee in the morning and afternoon, and return at the same time.

On arrival in Milwaukee, delegates and members are requested to register at once at the registration headquarters in the Hotel Pfister building, corner Wisconsin and Jefferson Streets. All are also requested to register ladies accompanying them on a separate register for the use of the Ladies' Reception and Entertainment Committee. The registration office will be open from 9 A. M. to 8 P. M. Monday, Tuesday, Wednesday and Thursday and from 9 A. M. to 1 P. M. Friday.

Telegrams will be forwarded by messengers to the stopping place in the city of the delegates and members if addressed "Care Headquarters A. M. A., Hotel Pfister." Letters must be called for at the headquarters' post-office.

As the city will be full of visitors at the time it is advised that all intending to attend the meeting should secure hotel or other accommodations in advance, for all will be required to register their place of abode in the city.

For any further information address the Chairman of the Committee of Arrangements, who will see that prompt attention is given to all communications.

U. O. B. Wingate, M.D.
*Chairman Committee of Arrangements.*

In the Section on Dental and Oral Surgery of the American Medical Association the following papers will be read:

Address, Dr. A. E. Baldwin.

Medication in Dental Practice, Dr. Edgar Palmer.

Caries and Necrosis, Dr. G. H. McCausey.

The Enemies of the Human Teeth, Dr. A. M. Benson.

Infection of the Mouth, Dr. E. L. Clifford.

Empyæma of the Maxillary Sinus, Dr. M. H. Fletcher.

Diseases of the Jaws, Dr. V. A. Latham.

Practical Therapeutics, Dr. George V. Brown.

Subject not given, Dr. Taft.

Fallacy of Foy's Theory of the Vault, Jaws, and Gums, being influenced by Temperament, Dr. E. S. Talbot.

### The National Association of Dental Faculties.

The annual meeting of this Body will be held in the World's Congress Art Palace, Chicago, beginning on Thursday, August 10th, at ten o'clock A. M , and continue probably through that and the succeeding day. It is important that all matters of business to come before that meeting be properly prepared beforehand, so that business can go promptly forward. It is to be hoped that all persons interested will give special attention to this request, and that every member be promptly present at the beginning of the meeting, as only the two days will be available for the work.

J. Taft.
Frank Abbott.
A. O. Hunt.
*Executive Committee.*

### The Annual Meeting of the California Dental Association.

The twenty-fourth annual meeting of this body will be held, commencing June 13th and continuing four days, in the Dental Department of the University of California.

This is one of the large, active, energetic State Societies of the country. An excellent programme has been prepared ; among the subjects to be presented are the following :

" Are these Few to be Saved," by Dr. G. A. Mills.

" Treating and Filling Pulpless Teeth," by E. A. Lundy, Honolulu.

" Ambidexterousness," by F. H. Metcalf.

" Dental Chips," by L. B. Holmes.

" Dental Art," by J. E. Cummings.

" Incidents in Extractions," by J. D. Hodgen.

" Conservative Dentistry," by F. W. Bliss.

" Ethics," by W. A. Knowles.

" The Study of Dental Anatomy, with Lantern Illustrations," by H. B. Carlton.

" Facial Neuralgia," by W. E. Nye.

One evening wil be wholly devoted to the Microscopic Committee, who will entertain the members and their friends with a Microscopic Soiree. F. O. Jacobs, Chairman.

Clinics will be held during the mornings of Wednesday, Thursday, and Friday, from nine to twelve.

There will be in addition a number of reports from standing committees on specified subjects.

This certainly constitutes a very interesting and attractive programme, one that ought to draw out all the better members of the profession in California.

Dr. W. Z. King, San Francisco, President.

Dr. L. Van Orden, San Francisco, Secretary.

Reduction in fares to members of the International Medical Congress, to be held in Rome, from September 24th to October 1st, 1893:

The North German Lloyd, 2 Bowling Green, N. Y., offers a reduction of 25 per cent. to members going to and coming from the 11th International Medical Congress, on Steamer Werra, which is to sail from New York on August 5th and September 9th, and on Steamer Fulda, on August 19th. Both these steamers sail to Genoa. The same reduction will be made for the return trips in October and November, on the same steamers, and for the Company's Saturday (off Bremen, Sunday off Southhampton,) steamers.

The Hamburg-American Packet Co., 37 Broadway, N. Y., 125 LaSalle Street, Chicago, offers a reduction of 25 per cent., both out and return, for all its steamers during the year 1893.

The Compagnie Generale Transatlantique, 3 Bowling Green, N. Y., offers the rates which are allowed French officers, that is, $63.50 for an $80 accommodation and $91.50 for a $120 accommodation.

Provision is also made for special reduced rates on the railways of the countries which the members of the Congress are to traverse.

### The Missouri State Dental Association.

The twenty-ninth annual meeting of the Missouri State Dental Association will be held at Excelsior Springs, Mo., July 11-12-13-14 inclusive. All dentists are invited to attend, as the meeting promises to be of great value to the profession.

Wm. Conrad,
321 N. Grand Av.
St. Louis, Mo.

### The World's Columbian Dental Congress—Special Notice.

To the Officers of Dental Societies in the United States and Foreign Countries.

Gentlemen: The Committees on Membership and Registration of the World's Columbian Dental Congress will be saved much trouble and the applicants for membership much vexation if the members of Dental Societies in good standing are furnished with credentials or certificates of membership so that they may be presented at the desk where intending members apply for their membership cards.

Advanced membership cards will be furnished on application to the Secretary of the General Executive Committee, or the Secretary General of the Congress when the membership fee ($10.00) accompanies the application.

A. O. Hunt,
Secretary of the General Executive Committee,
Iowa City, Iowa.

A. W. Harlan,
Secretary General of the Congress.
1000 Masonic Temple,
Chicago, Ills.

### EDITORIAL.

### THE DENTAL REGISTER,

A MONTHLY MAGAZINE DEVOTED TO THE INTERESTS OF THE

**DENTAL PROFESSION.**

*Terms Reduced to* $2.00 *Per Year. Single Copies,* 25 *Cents.*

**EDITED BY PROF. J. TAFT, D.D.S.**

—AND—

**Published by SAM'L A. CROCKER & CO., Ohio Dental and Surgical Depot.**

The volume commences in January and closes in December.

The REGISTER being issued monthly is always fresh, therefore Indispensable to the practitioner who desires to keep posted up with the new inventions and improvements which are daily developed. Neither expense nor effort will be spared to give value and variety to its contents. Articles will be illustrated with well executed engravings whenever they will add to the interest or assist to explanation.

Its extensive circulation and frequency of issue make it one of the BEST DENTAL ADVERTISING MEDIUMS in the United States.

All subscriptions must begin with January or July.

Local or special notices, five lines or less, will be inserted for $3 each insertion; over five lines, 50 cts. per line.

All advertising bills payable in advance, or at furthest, after the issue of the first number containing the advertisement. All transient advertisements to be paid for in advance.

☞CONTRIBUTIONS SOLICITED.

Exclusively Editorial Communications should be addressed to the Editor.

The latest number of the REGISTER may be ordered with or without other good at any time, and all letters should be addressed to

